# The I148M *PNPLA3* variant mitigates niacin beneficial effects: How the genetic screening in non-alcoholic fatty liver disease patients gains value

**DOI:** 10.3389/fnut.2023.1101341

**Published:** 2023-03-02

**Authors:** Erika Paolini, Miriam Longo, Marica Meroni, Giada Tria, Annalisa Cespiati, Rosa Lombardi, Sara Badiali, Marco Maggioni, Anna Ludovica Fracanzani, Paola Dongiovanni

**Affiliations:** ^1^General Medicine and Metabolic Diseases, Fondazione IRCCS Ca’ Granda Ospedale Maggiore Policlinico, Milan, Italy; ^2^Department of Pharmacological and Biomolecular Sciences, Università Degli Studi di Milano, Milan, Italy; ^3^Department of Clinical Sciences and Community Health, Università Degli Studi di Milano, Milan, Italy; ^4^Department of Pathophysiology and Transplantation, Università Degli Studi di Milano, Milan, Italy; ^5^Department of Surgery, Fondazione IRCCS Ca’ Granda Ospedale Maggiore Policlinico, Milan, Italy; ^6^Department of Pathology, Fondazione IRCCS Ca’ Granda Ospedale Maggiore Policlinico, Milan, Italy

**Keywords:** niacin, *PNPLA3* I148M, nutrigenomic, NAD, dietary supplementation

## Abstract

**Background:**

The PNPLA3 p.I148M impact on fat accumulation can be modulated by nutrients. Niacin (Vitamin B3) reduced triglycerides synthesis in *in vitro* and *in vivo* NAFLD models.

**Objectives:**

In this study, we aimed to investigate the niacin-I148M polymorphism crosstalk in NAFLD patients and examine niacin’s beneficial effect in reducing fat by exploiting hepatoma cells with different *PNPLA3* genotype.

**Design:**

We enrolled 172 (Discovery cohort) and 358 (Validation cohort) patients with non-invasive and histological diagnosis of NAFLD, respectively. Dietary niacin was collected from food diary, while its serum levels were quantified by ELISA. Hepatic expression of genes related to NAD metabolism was evaluated by RNAseq in bariatric NAFLD patients (*n* = 183; Transcriptomic cohort). Hep3B (148I/I) and HepG2 (148M/M) cells were silenced (siHep3B) or overexpressed (HepG2^I148^+^^) for *PNPLA3*, respectively.

**Results:**

In the Discovery cohort, dietary niacin was significantly reduced in patients with steatosis ≥ 2 and in I148M carriers. Serum niacin was lower in subjects carrying the G at risk allele and negatively correlated with obesity. The latter result was confirmed in the Validation cohort. At multivariate analysis, the I148M polymorphism was independently associated with serum niacin, supporting that it may be directly involved in the modulation of its availability. siHep3B cells showed an impaired NAD biosynthesis comparable to HepG2 cells which led to lower niacin efficacy in clearing fat, supporting a required functional protein to guarantee its effectiveness. Conversely, the restoration of PNPLA3 Wt protein in HepG2^I148^+^^ cells recovered the NAD pathway and improved niacin efficacy. Finally, niacin inhibited *de novo lipogenesis* through the ERK1/2/AMPK/SIRT1 pathway, with the consequent *SREBP1*-driven *PNPLA3* reduction only in Hep3B and HepG2^I148M+^ cells.

**Conclusions:**

We demonstrated a niacin-PNPLA3 I148M interaction in NAFLD patients which possibly pave the way to vitamin B3 supplementation in those with a predisposing genetic background.

## Introduction

Non-alcoholic fatty liver disease (NAFLD) is the chronic liver disorder with the highest prevalence worldwide whose pathogenesis is mainly related to the presence of obesity and type 2 diabetes (T2D). NAFLD comprises a plethora of clinical conditions ranging from simple steatosis to necroinflammation and ballooning which together define non-alcoholic steatohepatitis (NASH) that in turn may evolve to fibrosis and then to cirrhosis and hepatocellular carcinoma (HCC).

The environment is not the only predisposing factor to NAFLD which has a strong hereditable component and we previously demonstrated that hepatic fat is the main driver of the progression to end-stage liver damages in genetically predisposed individuals (1). In the last decade, the rs738409 (p.I148M; c.C444G) missense variation in *Patatin-like phospholipase domain-containing 3* (*PNPLA3*) has been consistently associated with the entire spectrum of NAFLD in different populations-based studies (2–4). PNPLA3 is the strongest genetic predictor of NAFLD, showing a high prevalence among NASH patients of whom 34% carry the mutant allele in homozygous. Heterozygous CG carriers have showed a prevalence of NASH and fibrosis higher than CC patients but lower than those carrying the GG genotype. Moreover, the homozygosity for PNPLA3 risk allele has been associated with more than 2-fold greater risk to develop NASH and cirrhosis, with up to a 12-fold increased risk for HCC and with an 18-fold increase in liver-related mortality (5, 6), thus suggesting a dose-dependent allele risk.

PNPLA3 protein localizes on lipid droplets (LD) surface and has triacylglycerol hydrolase and acyltransferase activities, which promote LDs remodeling in hepatocytes and hepatic stellate cells (7). The 148M substitution leads to a reduced fatty acid hydrolysis yielding an impaired mobilization of triglycerides (TG) which accumulate in the liver (8, 9). It has been showed that the methionine-isoleucine substitution at residue 148 in the PNPLA3 protein does not impact on the orientation of the catalytic dyad. However, the longer side chain of methionine blocks the access of fatty acids to the catalytic site, thus hampering PNPLA3 hydrolytic activity (10). Kumari et al. demonstrated that the I148M polymorphism increases the hepatic lipogenic activity and TG synthesis (7). Consistently, in a murine model, the overexpression of the mutated PNPLA3 (I148M) in the liver increased fatty acids and TG formation, impaired TAG hydrolysis and weakened the remodeling of TAG long-chain polyunsaturated fatty acids (PUFAs) (11). Moreover, it has been demonstrated that I148M variant disrupts ubiquitylation and proteasomal degradation of PNPLA3 thus resulting in the accumulation of mutated protein on lipid droplets and impaired lipids mobilization (12). Finally, it has been shown that carbohydrates may induce the accumulation of mutant PNPLA3 on LD surface thus worsening hepatic fat content (13).

To date there are no approved pharmacological treatments for the management of NAFLD and the clinical recommendations rely on lifestyle changes including daily exercise and healthy diet. Several drugs (anti-inflammatory, anti-fibrotic agents, and metabolic modulators), partially improving NASH activity and fibrosis, are under investigations even though they have produced marginally positive results (14). Although advanced stages of NAFLD are irreversible, isolated hepatic steatosis and early NASH offer a “therapeutic window” for targeted interventions based on nutritional and lifestyle modifications. Indeed, effective and sustained weight loss has been associated with marked improvement in glycemic control, hepatic insulin sensitivity, transaminases and liver histology (15).

It has been demonstrated that the response to diet may differ accordingly to the individual genetic background (15). A crosstalk between *PNPLA3* rs738409 variant and nutrition has been already assessed. A nutrigenetic analysis revealed that the hepatic fat fraction in GG carriers is strongly influenced by carbohydrates and dietary sugar whose consumption may induce sterol-regulatory element binding protein-1C (SREBP1c) and, in turn, the expression of PNPLA3 mutated protein thus exacerbating fat deposition (16). In addition, hepatic fat accumulation can be modulated by the interaction between PNPLA3 I148M variant and dietary omega 6/omega 3 PUFAs and diet supplemented with *n*-3 respect to *n*-6 PUFA could provide a targeted therapy in NAFLD subjects who are homozygous for the *PNPLA3* G allele (17).

It has also been demonstrated that a higher intake of several micronutrients including niacin (nicotinic acid or vitamin B3) could modulate hepatic steatosis. Pharmacological doses of niacin have favorable effects on lipid parameters, increasing high-density lipoprotein cholesterol (HDL-C) and decreasing low-density lipoprotein cholesterol (LDL-C), TG and lipoprotein (a) (18). Niacin administration in Sprague–Dawley rats fed high-fat diet significantly reduced hepatic and serum TG, lipid peroxidation thus ameliorating steatosis (19). In HepG2 cells treated with palmitic acid (PA) to mimic steatosis, niacin supplementation reduced the expression of *acyl-CoA diacylglycerol acyltransferase 2 (DGAT2)* responsible for the committing step of TG synthesis, the production of ROS and inflammation by inhibiting interleukin-8 (IL-8) (20). Li et al. revealed that *DGAT2* inhibition reduced nuclear localization of *SREBP1*c, which is involved in *de novo* lipogenesis (DNL) and in the transcriptional regulation of *PNPLA3*, thus providing a possible mechanism through which niacin could ameliorate hepatic fat accumulation (21). Additionally, niacin may improve steatosis by inhibiting DNL through the activation of ERK1/2/AMPK/SIRT1 pathway. SIRT1 is involved in the transcriptional regulation of *SREBP1c* paralleling the epigenetic regulation of *PNPLA3* gene and together with the NAD driven-AMPK activation downregulate DNL, suggesting a possible gene-nutrient interplay and providing another strategy across which niacin could exert its beneficial role (22, 23).

Consistently, patients with hypertriglyceridemia and treated with niacin (Niacin ER, trade name: Niaspan) showed a significant reduction of liver and visceral fat whereas the *DGAT2* rs3060 and rs101899116 variants were associated with a smaller decrease in liver fat content in response to niacin (24). Finally, Linder et al. evaluated whether dietary niacin intake predicts change of liver fat content during a lifestyle intervention. Among fat compartments, the hepatic one showed the largest decrease and about half of NAFLD patients reached a steatosis resolution thus suggesting that niacin-fortified foods may represent a valuable strategy to treat the disease, taking into consideration the individual genetic background (25).

Therefore, this study aimed to assess the dietary and circulating niacin levels in NAFLD patients stratified according to the presence of the I148M variant which represents the stronger genetic predictor related to steatosis whose impact on fat deposition may be modulated by nutrients. Moreover, we examined the efficacy of niacin in reducing fat accumulation by exploiting Hep3B and HepG2 cells, which are wild-type and homozygous for the I148M mutation, respectively.

## Materials and methods

### Discovery cohort

We enrolled patients affected by NAFLD (*n* = 172; Discovery cohort), of whom the presence of steatosis was non-invasively evaluated through ultrasound echography using a convex 3.5 MHz probe and by FibroScan^®^, at the Fondazione IRCCS Cà Granda Ospedale Maggiore Policlinico, Milano. FibroScan^®^ is a rapid and painless ultrasound (US)-based technique which emits low-frequency (50 mHz) vibrations into the liver, creating a propagating shear wave. This is detected by a pulse-echo acquisition that calculates its velocity which, in turn, is proportional to the stiffness of the tissue passed through. The Controlled attenuation parameter (CAP) value ≥ 248 estimated the presence of liver steatosis, whereas the liver stiffness measurement (LSM) value ≥ 7.0 and ≥ 6.2 kPa defined a significant liver fibrosis (26, 27). Patients were genotyped to assess the presence of the rs738409 C > G (p.I148M) *PNPLA3* variant as previously described (28–30), and the population was consistent with Hardy-Weinberg equilibrium (*p* = 0.13). Moreover, in order to assess dietary habits, we requested patients to carefully compile a food diary for 3 weeks. Kilocalories (kcal) of micro- and macro-nutrients were calculated with MètaDieta software^1^ and are listed in Supplementary Table 1. Dietary products containing niacin are shown in Supplementary Table 2. Demographic, anthropometric and clinicopathological features of the Discovery cohort are shown in Supplementary Table 3.

### Validation cohort

The Validation cohort includes 358 NAFLD unrelated patients of European descent who were consecutively enrolled at the Metabolic Liver Diseases outpatient service at Fondazione IRCCS Cà Granda, Ospedale Maggiore Policlinico, Milan, Italy. Inclusion criteria were the availability of liver biopsies performed for suspected NASH or severe obesity, DNA samples, and clinical data. Individuals with excessive alcohol intake (men, > 30 g/day; women, > 20 g/day), viral and autoimmune hepatitis, or other causes of liver disease were excluded. Patients were stratified according to both the presence of the rs738409 C > G (p.I148M) *PNPLA3* variant and the population was not consistent with Hardy-Weinberg equilibrium (*p* = 0.03). The study conformed to the Declaration of Helsinki and was approved by the Institutional Review Board of the Fondazione Ca’ Granda IRCCS of Milan and relevant Institutions. All participants gave written informed consent. Patients’ genotyping and histological evaluation are presented in the Supplementary materials and methods. Demographic, anthropometric and clinicopathological features of the Discovery cohort are shown in Supplementary Table 4.

### Transcriptomic cohort

RNA-seq was performed in a subset of 183 severely obese patients (31 without and 152 with NAFLD) in whom a percutaneous liver biopsy was performed during bariatric surgery at Fondazione IRCCS Cà Granda, Ospedale Policlinico, Milan, Italy (31). The study was conformed to the Declaration of Helsinki and approved by the Institutional Review Boards and their Ethics Committees. All participants gave written informed consent. Clinical characteristics of patients of whom RNA-seq data was available are presented in Supplementary Table 5. RNA-seq mapping descriptive statistics, detailed protocol, data analysis approach, patients’ genotyping and histological assessment are described in the Supplementary materials and methods.

### Measurement of circulating niacin

Niacin levels were evaluated in sera of both Discovery (*n* = 172) and Validation (*n* = 358) cohorts, collected at the time of NAFLD diagnosis. Niacin was quantified through the “ID-Vit Niacin” assay (Immunodiagnostik AG., Germany), based on a microbiological method which measures the total free niacin contained in the serum. The assay exploits microtiter plates covered with *Lactobacillus plantarum*, which uses niacin to grow. Serum samples were incubated at 37°C for 48 h and then turbidity was measured at λ = 610–630 nm with a spectrophotometer. The amount of niacin in the serum was directly proportional to the bacterial growth.

### *In vitro* models and treatments

To investigate the possible interaction between *PNPLA3* and niacin metabolism, we compared two human hepatoma cell lines with a different genetic background (Hep3B, HepG2) and commonly used to study liver metabolism *in vitro*. The Hep3B cells are wild-type for the *PNPLA3* gene, while the HepG2 cells carry the rs738409 C > G (I148M) *PNPLA3* polymorphism in homozygosity. Both cell lines were cultured in Dulbecco’s modified eagle’s medium (DMEM) containing 10% fetal bovine serum (FBS), 100 U/L penicillin, 100 U/L streptomycin and 1% L-glutamine (Life Technologies-ThermoFisher Scientific, Waltham, MA, USA) and maintained at 37°C and 5% CO_2_. To mimic human steatosis, both cell types were exposed to PA at 0.25 mM, whereas to assess the niacin efficacy on fat accumulation in hepatocytes, they were supplemented with a mixture of PA and niacin (PA + NIA) at 0.5 mM for 24 hours. Treatments were freshly prepared and administered when appropriate.

### RNA interference

Hep3B cells were transiently transfected for 48 h by pooling three different target-specific siRNA oligo duplexes directed against the exons 2, 4, and 5 of the human *PNPLA3* (siHep3B) in order to improve the gene-silencing efficiency and at a final concentration of 10 μM (MyBioSource, Inc., San Diego, CA, USA). Cyclophilin B (10 μM) was used as a scramble negative control (Horizon Discovery, Waterbeach, UK).

### *PNPLA3* llentiviral overexpression

*PNPLA3* was stably overexpressed in HepG2 cells through pLenti-C-mGFP lentiviral vector which were engineered to express a complete Open Reading Frame (ORF) fused with green fluorescent protein (GFP) tag (henceforth HepG2^I148^+^^). We seeded 3 × 10^4^ cells in 24-well plates and incubated at 37^°^C and 5% CO_2_ overnight. Multiplicity of infection (MOI) was set at 2.5 and the amount of lentiviral particles for the transduction were calculated according to the manufactures‘instruction (OriGene, Rockville, USA). Lentiviral particles were added to pre-warmed cultured media for 24 h. To introduce PNPLA3-GFP tagged protein, HepG2 cells were transduced with *PNPLA3* Human Tagged ORF Clone Lentiviral Particle (OriGene, Rockville, USA) containing a molecular sequence which aligns with the PNPLA3 mRNA (gene accession number: NM_025225).

### Statistical analysis

For descriptive statistics, continuous variables were reported as mean and standard deviation or median and interquartile range for highly skewed biological variables. Variables with skewed distribution were logarithmically transformed before analysis. Differences between two groups were calculated with non-parametric Wilcoxon test, followed by *post hoc* pairwise comparison. One-way non-parametric ANOVA (Kruskal–Wallis) followed by *post hoc* Dunn’s multiple comparison test was used to compare multiple groups adjusted for the number of comparisons. *P*-values < 0.05 were considered statistically significant. Statistical analyses were performed using JMP 16.0 (SAS, Cary, NC) and Prism software (version 6, GraphPad Software).

## Results

### The *PNPLA3* I148M variant modulates both dietary and serum niacin levels in NAFLD patients and more so in the presence of obesity

Genetic variations may modulate the response to therapeutic approaches and vitamins in NAFLD patients. Among the latter, niacin has been reported to protect against severe steatosis although its interplay with genetics remains to be elucidated. The assessment of food diary in 172 patients with a non-invasive assessment of NAFLD revealed that niacin was the micronutrient with the lowest dietary levels in carriers of the PNPLA3 I148M variation (*p* = 0.04 CG/GG vs CC; Supplementary Table 1). Therefore, in the attempt to investigate the impact of the rs738409 C > G *PNPLA3* genotype on niacin metabolism, we assessed the vitamin absorption in the serum of NAFLD patients belonging to the Discovery cohort. Alimentary and circulating niacin levels were then correlated with clinical-pathological features of NAFLD subjects in order to evaluate a possible gene-environment interaction.

**FIGURE 1:**
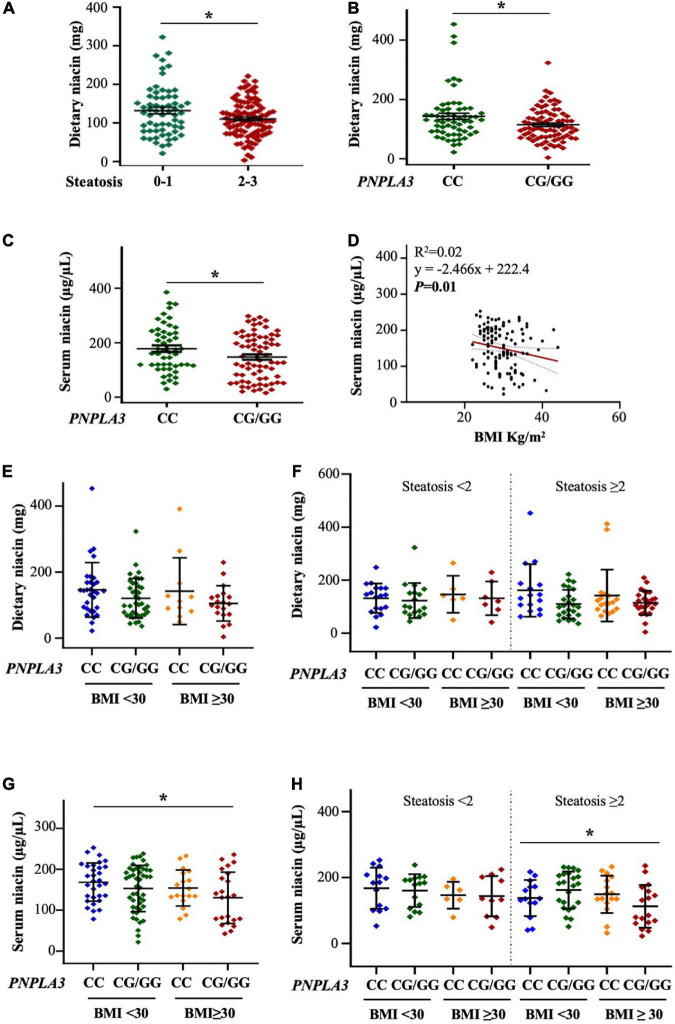
The PNPLA3 I148M variant affects alimentary and serum niacin levels in the discovery cohort. **(A,B)** Niacin intake was reduced in NAFLD patients with steatosis ≥ 2 and *PNPLA3* CG/GG mutation at bivariate analysis (*p < 0.05 at Wilcoxon test, *vs* steatosis < 2 and *vs PNPLA3* CC). **(C)** Circulating niacin levels were lower in PNPLA3 I148M carriers at bivariate analysis compared to non-carriers (*p < 0.05 at Wilcoxon test). **(D)** Negative correlation between serum niacin concentration and body mass index (BMI). **(E,F)** Bivariate analysis shows the trend of dietary niacin intake in NAFLD patients stratified according to both the *PNPLA3* G at-risk allele and obesity. In panel **(F)**, NAFLD patients were even subcategorized by the presence of steatosis < 2 and steatosis ≥ 2. **(G,H)** The *PNPLA3* at-risk genotype affected niacin absorption in a subgroup of obese NAFLD subjects and with steatosis ≥ 2 (*p* = 0.005 at ANOVA, *p < 0.05 *vs* BMI < 30 CC; *p* = 0.04 at ANOVA, *p < 0.05 *vs* steatosis ≥ 2 with BMI < 30 CC).

In the Discovery cohort, 114/172 patients (66.27%) had severe steatosis (grade 2-3) and 104/172 (60.46%) carried the *PNPLA3* p.I148M variant (Supplementary Table 3). At bivariate analysis, niacin intake was lower in NAFLD subjects with steatosis ≥ 2 compared to those with low-grade or no steatosis (*p* < 0.05 at Wilcoxon, adj *p* = 0.02 *vs* steatosis < 2, Figure 1A) and in *PNPLA3* CG/GG carriers (*p* < 0.05 at Wilcoxon, adj *p* = 0.02 vs *PNPLA3* CC, Figure 1B).

Similarly, serum niacin was reduced in individuals carrying the *PNPLA3* CG/GG mutation (*p* < 0.05 at Wilcoxon, adj *p* = 0.03 *vs PNPLA3* CC, Figure 1C) although no significant differences in its levels emerged among patients with steatosis < 2 and ≥ 2 (Supplementary Figure 1A), possibly suggesting that the presence of the rs738409 C > G *PNPLA3* genotype rather than hepatic fat accumulation may influence niacin absorption or metabolism. Moreover, at correlation analysis, serum niacin negatively associated with body mass index (BMI) (*p* < 0.01, Figure 1D) supporting the hypothesis that niacin availability may be even swayed by environmental risk factors as obesity.

Therefore, to deepen the impact of obesity on niacin metabolism, we stratified the Discovery cohort according to BMI ≥ 30 kg/m^2^ and to the presence of the I148M variant. We found that dietary niacin showed a trend of reduction in patients carrying the *PNPLA3* at-risk G allele and BMI ≥ 30 kg/m^2^ (Figure 1E) whereas by stratifying patients according to the presence of severe steatosis the lower levels of niacin in subjects with the *PNPLA3* CG/GG mutation didn’t vary across BMI (Figure 1F). We next assessed circulating niacin and we observed that its serum levels tended to be lower in G allele carriers and the reduction became significant in those with BMI ≥ 30 kg/m^2^ (*p* = 0.005 at ANOVA, adj *p* = 0.03 *vs* BMI < 30 CC, Figure 1G). This effect was highly emphasized in the subcategory of patients with steatosis ≥ 2 (*p* = 0.04 at ANOVA, adj *p* = 0.03 *vs* steatosis ≥ 2 with BMI < 30 CC, Figure 1H), probably due to the additive weight of PNPLA3 I148M variant and obesity in niacin absorption.

At generalized linear model adjusted for sex, age, steatosis ≥ 2 and alimentary niacin, the presence of the *PNPLA3* CG/GG mutation was independently associated with serum niacin in NAFLD individuals with BMI ≥ 30 kg/m^2^ (β = –0.21, 95% CI: –0.41–0.004, *p* = 0.04, Table 1), thereby supporting that PNPLA3 p.I148M aminoacidic substitution may be directly involved in the alteration of systemic niacin availability and that the effect attributable to this mutation may be amplified by adiposity.

**TABLE 1:** Association among serum niacin (μg/μL) levels and the *PNPLA3* rs738409 C > G (p.I148M) variant in the discovery cohort (*n* = 172), stratified according to BMI ≥ 30 kg/m^2^.

	Circulating niacin (μ g/μ L)					
	BMI < 30			BMI ≥ 30		
	β	95% CI	*P*-value*	β	95% CI	*P*-value*
Sex, M	–0.02	–0.14 to 0.10	0.73	0.03	–0.19 to 0.26	0.74
Age, years	–0.006	–0.014 to 0.002	0.15	0.0002	–0.01 to 0.01	0.97
Steatosis ≥ 2, yes	–0.03	–0.12 to 0.07	0.57	–0.12	–0.31 to 0.07	0.21
Dietary niacin (kcal)	–0.12	–0.29 to 0.04	0.15	–0.19	–0.44 to 0.05	0.12
*PNPLA3* G allele, yes	–0.03	–0.13 to 0.06	0.46	–0.21	–0.41 to 0.004	**0.04**

Analysis was performed in 172 NAFLD patients stratified according to body mass index (BMI) ≥ 30 kg/m^2^, of whom the serum niacin dosage was available. Bold values were obtained at generalized linear model adjusted for sex, age, steatosis ≥ 2, dietary niacin and the presence of *PNPLA3* G allele. Both alimentary and circulating niacin levels were log-transformed. Serum niacin was considered as an independent variable. CI, confidence interval. *Statistically significant relationship between each predictor variable and the response variable in the linear regression analysis.

### The additive effect of the *PNPLA3* I148M mutation and obesity impacts on circulating niacin in patients with biopsy-proven NAFLD

**FIGURE 2:**
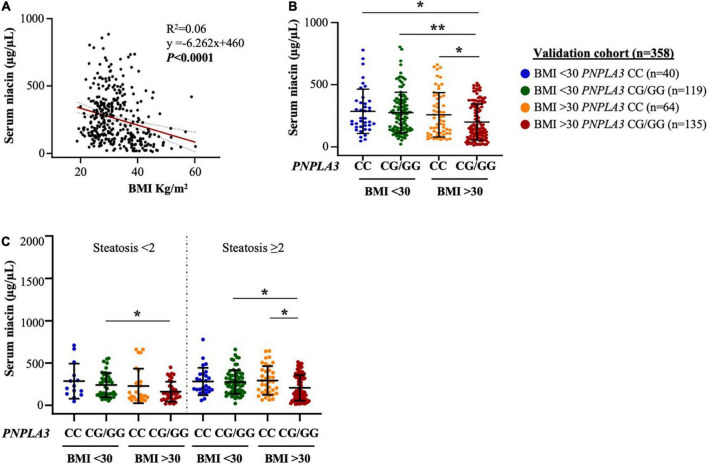
Obesity heightens PNPLA3 genetic risk on niacin absorption in the validation cohort. **(A)** Negative correlation between serum niacin concentration and body mass index (BMI). **(B,C)** The presence of the PNPLA3 I148M variant combined with obesity reduced niacin absorption in biopsied NAFLD subjects, independently of steatosis severity (*p* = 0.004 at ANOVA, *p < 0.05 *vs PNPLA*3 CG/GG with/without obesity and *vs PNPLA3* CC with BMI ≥ 30). *p* = 0.0001 at ANOVA, **p < 0.01 *vs. PNPLA3* CG/GG without obesity.

To validate the results obtained in the Discovery cohort, we measured serum niacin in a larger cohort including *n* = 358 subjects with histological assessment of NAFLD, of whom dietary niacin was not available (Validation cohort). In this cohort, steatosis ≥ 2 was diagnosed in 232/358 patients (64.80%), whereas obesity (BMI ≥ 30 kg/m^2^) and the PNPLA3 I148M variant were identified in 194/358 (54.18%) and 255/358 (71.2%), respectively (Supplementary Table 4). Circulating niacin concentration was inversely correlated with BMI (*p* < 0.0001, Figure 2A) and the lowest levels were observed in individuals affected by obesity and carrying the *PNPLA3* CG/GG mutation thus confirming the results obtained in the Discovery cohort (*p* = 0.0001 at ANOVA, adj *p* < 0.05 *vs PNPLA3* CC with either BMI < 30 or BMI ≥ 30; adj *p* < 0.01 *vs PNPLA3* CG/GG with BMI < 30, Figure 2B).

Moreover, serum niacin levels were decreased in presence of both obesity and the *PNPLA3* G risk allele, thereby resembling what observed in the Discovery cohort although we didn’t observe any variance between patients with steatosis < 2 and ≥ 2 (*p* = 0.004 at ANOVA, adj *p* < 0.05 *vs PNPLA3* CG/GG with/without obesity and *vs PNPLA3* CC with BMI ≥ 30, Figure 2C). In addition, no relevant differences were found in serum niacin levels by stratifying patients according to the severity of necroinflammation, fibrosis and NAS (Supplementary Figures 1B–D), suggesting that whole spectrum of NAFLD *per se* may not influence niacin absorption.

At multivariate analysis adjusted for sex, age and steatosis ≥ 2 the association between the *PNPLA3* I148M variant and lower serum niacin remained strongly significant in NAFLD individuals who belonged to the Validation cohort with BMI ≥ 30 kg/m^2^ (β = –0.24, 95% CI: –0.43–0.06, *p* = 0.009, Table 2), thus corroborating the hypothesis that the *PNPLA3* p.I148M missense variation may be a genetic modifier of vitamin B3 metabolism.

**TABLE 2:** Association between serum niacin (μg/μL) levels and the *PNPLA3* rs738409 C > G (p.I148M) variant in the validation cohort (*n* = 358), stratified according to BMI ≥ 30 kg/m^2^.

	Circulating niacin (μg/μL)					
	BMI < 30			BMI ≥ 30		
	β	95% CI	*P*-value*	β	95% CI	*P*-value*
Sex, M	–0.02	–0.11 to 0.06	0.60	–0.02	–0.22 to 0.17	0.80
Age, years	0.0001	–0.006 to 0.007	0.96	0.0002	–0.01 to 0.015	0.97
Steatosis ≥ 2, yes	0.07	–0.009 to 0.16	0.08	–0.09	–0.26 to 0.08	0.29
*PNPLA3* G allele, yes	–0.02	–0.11 to 0.06	0.56	–0.24	–0.43 to 0.06	**0.009**

Analysis was performed in 358 biopsied patients stratified according to BMI ≥ 30 kg/m^2^, of whom the serum niacin dosage was available. Bold values were obtained at generalized linear model adjusted for sex, age, steatosis ≥ 2 and the *PNPLA3* G allele, yes. The circulating niacin was log-transformed and considered as an independent variable. CI, confidence interval. *Statistically significant relationship between each predictor variable and the response variable in the linear regression analysis.

### The *PNPLA3* I148M variation modulates hepatic enzymes of NAD metabolism in NAFLD patients

The results obtained in the Discovery and Validation cohorts have suggested that niacin availability, the primary source of NAD synthesis, may be affected in patients with the *PNPLA3*-driven genetic predisposition to develop NAFLD and more so in obese ones. In order to evaluate whether the presence of the *PNPLA3* I148M variant may even interfere with hepatic NAD metabolism, we assessed the expression of genes involved in NAD biosynthetic pathways as well as NAD/NADH-dependent enzymes through RNA-seq analysis performed in 183 biopsied NAFLD patients (Transcriptomic cohort) who underwent bariatric surgery.

**FIGURE 3:**
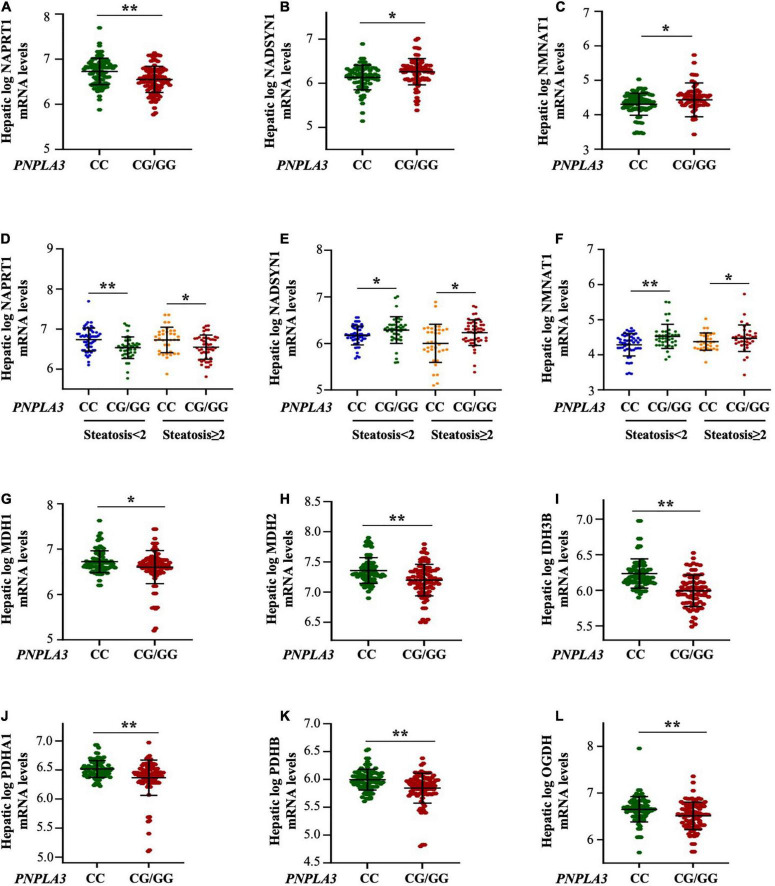
The PNPLA3 G allele impairs hepatic NAD metabolism in the transcriptomic cohort independently of steatosis severity. **(A–C)** Hepatic *NAPRT1* mRNA levels were reduced in the presence of the *PNPLA3* CG/GG mutation (**p < 0.01 at Wilcoxon test *vs PNPLA3* CC), while *NADSYN1* and *NMNAT1* expression were upregulated in *PNPLA3* CG/GG carriers (*p < 0.05 at Wilcoxon test *vs PNPLA3* CC). **(D–F)** Hepatic *NAPRT1, NADSYN1*, and *NMNAT1* expression were modulated in response to *PNPLA3* CG/GG mutation, but regardless of steatosis grade (NAPRT1: *p* = 0.0004 at ANOVA, **p < 0.01 *vs PNPLA3* CC with steatosis < 2; *p < 0.05 *vs PNPLA3* CC with steatosis ≥ 2; NADSYN: *p* = 0.0001 at ANOVA, *p < 0.05 vs *PNPLA3* CC with steatosis < 2 and ≥ 2; NMNAT1: *p* = 0.0001 at ANOVA, **p < 0.01 *PNPLA3* CC with steatosis < 2, *p < 0.05 *vs PNPLA3* CC with ≥ 2). **(G–L)** The mRNA levels of NAD-utilizing complexes (MDH1/2. IDH3B, PDHA1/B and OGDB) were decreased in presence of the *PNPLA3 G* at-risk allele (*p < 0.05 and **p < 0.01 at Wilcoxon *vs PNPLA3* CC).

At bivariate analysis, the hepatic *nicotinate phosphoribosyl-transferase 1* (*NAPRT1*) mRNA levels, the main enzyme involved in niacin conversion into NAD precursors, were lower in carriers of the *PNPLA3* G allele compared to wild-type group (*p* < 0.01 at Wilcoxon, adj *p* = 0.0026 *vs PNPLA3* CC, Figure 3A). Conversely, the expression of *NAD synthetase 1* (*NADSYN1*) and *nicotinamide phosphoribosyl-transferase* (*NMNAT1*), alternatively producing NAD from tryptophan and the salvage pathway, was increased in I148M *PNPLA3* carriers compared to non-carriers (*p* < 0.05 at Wilcoxon, adj *p* = 0.01 and adj *p* = 0.03,*vs PNPLA3* CC, respectively, Figures 3B, C) possibly due to a compensatory mechanism to provide NAD in the liver.

Similarly to what observed in the Discovery and Validation cohorts stratified according to steatosis grade, the mRNA levels of *NAPRT1*, *NADSYN1* and *NMNAT1* were not affected by the severity of steatosis (*p* = 0.0004 at ANOVA, adj *p* = 0.003 and *p* = 0.02 *PNPLA3* CG/GG with steatosis < 2 and ≥ 2 *vs PNPLA3* CC with steatosis < 2 and ≥ 2, respectively; Figures 3D; P = 0.0001 at ANOVA, adj *p* = 0.004 and *p* = 0.03 *PNPLA3* CG/GG with steatosis < 2 and ≥ 2 *vs PNPLA3* CC with steatosis < 2 and ≥ 2, respectively Figures 3E, F), supporting that the *PNPLA3* genetic variant more than hepatic fat accumulation influences the niacin-dependent NAD metabolism.

Moreover, the hepatic mRNA levels of the NAD/NADH-dependent enzymes as the *malate dehydrogenase 1/2* (*MDH1/2*), the *isocitrate dehydrogenase [NAD] subunit beta* (IDH3B), the *pyruvate dehydrogenase E1 subunits alpha A1 and beta* (*PDHA1/B*) and the *oxoglutarate dehydrogenase* (*OGDH*) were significantly reduced in carriers of the *PNPLA3* G allele (adj *p* < 0.05 and adj *p* = 0.0001 at Wilcoxon *vs PNPLA3* CC genotype, Figures 3G–L), suggesting that the rs738409 C > G *PNPLA3* at-risk genotype may affect vitamin B3 metabolism by modulating the expression of NAD/NADH-consuming genes.

### The *PNPLA3* loss-of-function impairs NAD metabolism in hepatoma cells

Evidence in NAFLD patients highlighted that niacin availability and the hepatic NAD biosynthesis are altered by the presence of the I148M polymorphism. Therefore, to explore the possible interaction between *PNPLA3* and niacin metabolism, we compared NAD biosynthetic rate in Hep3B and HepG2 cells, which carried the *PNPLA3* CC and GG genotype, respectively. Moreover, we induced the *PNPLA3* silencing in Hep3B cells (siHep3B) in order to evaluate whether its loss-of-function may impair NAD production. Finally, we overexpressed the *PNPLA3* Wt gene (HepG2^I148^+^^) in HepG2 cells attempting to elucidate whether the re-introduction of the *PNPLA3* Wt form may restore vitamin B3 efficacy in reducing fat accumulation.

**FIGURE 4:**
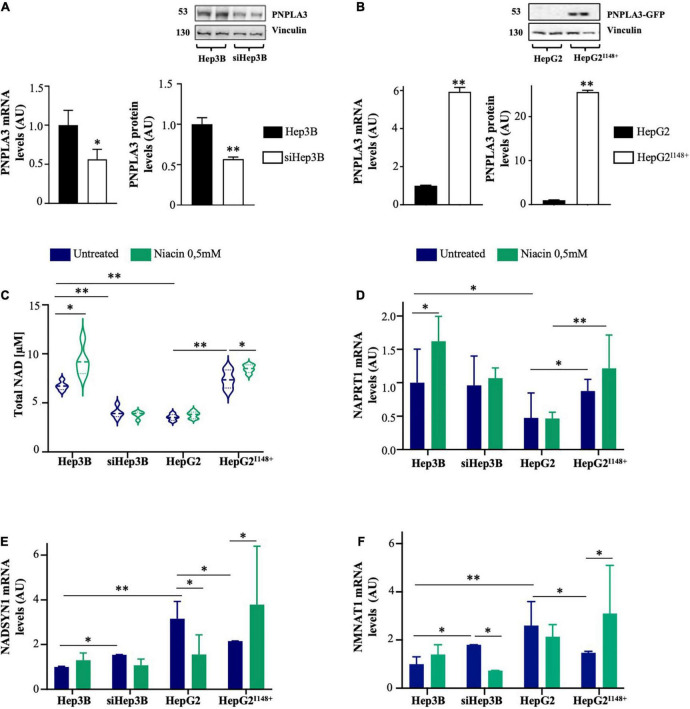
PNPLA3 loss-of-function dampens NAD synthesis in hepatocytes after niacin administration. **(A,B)** PNPLA3 mRNA and protein levels were assessed by qRT-PCR and Western blot, respectively, in hepatoma cells. **(C)** NAD content was assessed in Hep3B, siHep3B, HepG2 and HepG2^I148^+^^ cells through NAD/NADH Colorimetric/Fluorometric Assay Kit at both baseline and after niacin exposure. **(D–F)** *NAPRT1, NADSYN* and *NMNAT1* mRNA levels evaluated by qRT-PCR in hepatoma cells (Hep3B, siHep3B, HepG2 and HepG2^I148^+^^) with or without niacin treatment. For gene expression, data were normalized to *ACTB* housekeeping gene and expressed as fold increase (Arbitrary Unit-AU) compared to control group. For Western blot, data were normalized to vinculin housekeeping protein and expressed as fold increase (AU) compared to control group. For violin plot, data were expressed as median concentration (thick dashed lines) and interquartile range (dotted lines). Adjusted *p < 0.05 and ^**^p < 0.01.

As expected, *PNPLA3* mRNA and protein levels were reduced in siHep3B by around 50% (*p* < 0.05 and *p* < 0.01 *vs* scramble, Figure 4A), while they were significantly increased after the lentiviral transduction in HepG2^I148^+^^ cells (*p* < 0.01 *vs* HepG2, Figure 4B).

At baseline, Hep3B cells showed higher NAD content compared to siHep3B ones (*p* < 0.01 *vs* siHep3B; Figure 4C), indicating that the *PNPLA3* deficiency may affect NAD production. Likewise, HepG2 cells exhibited lower NAD concentration, matching the levels measured in siHep3B cells, whereas in HepG2^I148^+^^ model it was comparable to Hep3B. After niacin exposure, NAD concentration increased in Hep3B cells but not in siHep3B ones (*p* < 0.05 *vs* Hep3B untreated, Figure 4C) supporting that *PNPLA3* silencing may modify the response to niacin supply. Furthermore, intracellular NAD content remain unchanged in HepG2 cells after niacin administration, showing a similar range of that observed in siHep3B ones, while it was increased in HepG2^I148^+^^ model (*p* < 0.05 *vs* HepG2^I148^+^^ untreated, Figure 4C).

Furthermore, in HepG2 cells the *NAPRT1* expression was reduced compared to both HepG2^I148^+^^ and Hep3B Wt models (*p* < 0.01 and *p* < 0.05 *vs* HepG2^I148^+^^, *p* < 0.01 and *p* < 0.05 *vs* Hep3B; Figures 4C, D), thereby sustaining that the *PNPLA3* I148M variation may be involved in the impairment of the canonical NAD synthesis. Consistently with the increased NAD content upon niacin treatment, *NAPRT1* expression was induced in Hep3B cells (*p* < 0.05 *vs* Hep3B untreated, Figure 4D), whereas it was not modified in siHep3B ones. Similarly, HepG2 cells didn’t show an increment of *NAPRT1* mRNA levels after niacin exposure, while its expression was slightly induced in the *PNPLA3* overexpressed cells, resembling the results obtained in Hep3B.

Moreover, both siHep3B and HepG2 cells showed higher basal expression of *NADSYN1* and *NMNAT1* than Hep3B and HepG2^I148^+^^ cells (*p* < 0.01 *vs* Hep3B; *p* < 0.05 *vs* HepG2^I148^+^^; Figures 4E, F), possibly to compensate NAD shortage and corroborating the results obtained in the Transcriptomic cohort.

As concern the alternative pathways of NAD biosynthesis, both Hep3B and HepG2^I148^+^^ displayed high *NADSYN1* and *NMNAT1* mRNA levels after niacin exposure (*p* < 0.05 *vs* HepG2^I148^+^^ untreated, Figures 4E, F). Contrariwise, siHep3B and HepG2 cells reduced the expression of *NADSYN1* and *NMNAT1* enzymes (*NADSYN1: p* < 0.05 *vs* HepG2 untreated, Figure 4E; *NMNAT1: p* < 0.01 *vs* siHep3B untreated, Figure 4F) probably due to a negative feedback loop exerted by niacin or its metabolites on the alternative pathways.

Thus, these findings may support that the presence of the *PNPLA3* loss-of-function induced by the silencing or the I148M variant may impair the canonical *via* of NAD biosynthesis at both baseline and after niacin supplementation. In support of this hypothesis, our *in vitro* results have suggested that the re-establishment of the PNPLA3 functional protein seems to rescue the vitamin B3 metabolism in hepatocytes, thereby sustaining the link between PNPLA3 and NAD availability.

### The *PNPLA3* loss-of-function mitigates the beneficial role of niacin in reducing triglycerides synthesis

It has been previously demonstrated that niacin improves hepatic steatosis by downregulating *DGAT2* and reducing TG synthesis which, in turn, lead to a decreased oxidative stress (19, 20). In order to evaluate whether the presence of the I148M polymorphism may disturb niacin effectiveness on intracellular fat content, we treated hepatoma cells with PA alone or combined with NIA for 24 h.

**FIGURE 5:**
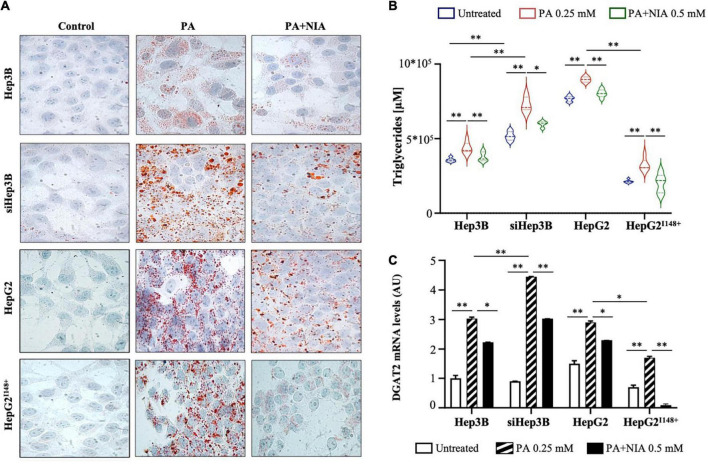
**(A)** Evaluation of LDs formation was assessed in hepatoma cells (Hep3B, siHep3B, HepG2, and HepG2^I148^+^^) after PA challenge and NIA treatment by ORO staining (100× magnification). Nuclei were counterstained by hematoxylin. **(B)** TG content was measured in cell lysates (Hep3B, siHep3B, HepG2, and HepG2^I148^+^^) through Triglycerides Colorimetric/Fluorometric Assay Kit. **(C)** *DGAT2* mRNA levels were quantified in hepatoma cells with or without niacin treatment after PA challenge (Hep3B, siHep3B, HepG2, and HepG2^I148^+^^) by qRT-PCR. For gene expression, data were normalized to *ACTB* housekeeping gene and expressed as fold increase (Arbitrary Unit-AU) compared to control group. For violin plot, data were expressed as median concentration (thick dashed lines) and interquartile range (dotted lines). Adjusted *p < 0.05 and ^**^p < 0.01.

At ORO staining, both Hep3B and HepG2^I148^+^^ cells accumulated less lipid droplets in response to PA administration rather than siHep3B and HepG2 cell lines, possibly due to the efficient PNPLA3 hydrolytic activity (Figure 5A). PA exposure enhanced the intracellular TG content and induced *DGAT2* upregulation in all experimental models (*p* < 0.01 *vs* Hep3B, siHep3B, HepG2 and HepG2^I148^+^^ untreated, Figures 5B, C). However, siHep3B and HepG2 cells exhibited an exacerbated lipid accumulation and *DGAT2* induction as a consequence of low lipid clearance induced by *PNPLA3* silencing or I148M variant, respectively (*p* < 0.01 and *p* < 0.05 *vs* Hep3B + PA or *vs* HepG2^I148^+^^ + PA, Figures 5B, C).

After niacin administration, lipid overload was reduced in all *in vitro* models, albeit this effectiveness was mitigated in siHep3B and HepG2 cells, supporting that the presence of a non-functional PNPLA3 protein may interfere with niacin protective role (Figure 5A). In keeping with the ORO staining, niacin administration strongly reduced the intracellular TG content (*p* < 0.01 *vs* Hep3B + PA, siHep3B + PA, HepG2 + PA and HepG2^I148^+^^ + PA, Figure 5B) and the mRNA levels of *DGAT2* in all cell lines (*p* < 0.05 *vs* Hep3B + PA and HepG2 + PA, *p* < 0.01 *vs* siHep3B and HepG2^I148^+^^ + PA, Figure 5C), showing the greatest effect in Hep3B and HepG2^I148^+^^ models (Figures 5B, C) and sustaining that the presence of PNPLA3 in the WT form may improve the niacin efficacy on fat overload clearance.

Consistently with the worsened fat accumulation, siHep3B and HepG2 cells showed higher ER-oxidative injury compared to those induced by PA in Wt cellular models, by increasing the mRNA levels of *Activating Transcription Factor 4-6 (ATF4*, *ATF6)* and *Glucose-regulated protein 78 (GRP78)* and enhancing the production of hydrogen peroxide (H_2_O_2_) and malondi- aldehyde (MDA) (*p* < 0.05 and *p* < 0.01 *vs* Hep3B + PA and HepG2^I148^+^^ + PA; Supplementary Figures 2A–E).

Moreover, we found that niacin treatment strongly counteracted the negative effects of PA on *ATF4* (*p* < 0.05 *vs* PA, *p* < 0.01 *vs* PA, Supplementary Figure 2A), *ATF6* (*p* < 0.05 *vs* PA, *p* < 0.01 *vs* PA, Supplementary Figure 2B), *GRP78* expression (*p* < 0.01 vs PA, Supplementary Figure 2C) and oxidative injury (H_2_O_2_: *p* < 0.05 *vs* PA, *p* < 0.01 *vs* PA, Supplementary Figure 2D; MDA: *p* < 0.05 *vs* PA, *p* < 0.01 *vs* PA, Supplementary Figure 2E) in all the *in vitro* models. Although these findings have suggested that the effect of niacin in reducing TG synthesis may differ accordingly to the *PNPLA3* genetic background in our experimental models, we could speculate that the impact on hepatocellular toxicity may be more a consequence of the reduced fat accumulation rather than dependent by PNPLA3.

### The *PNPLA3* loss-of-function promotes *de novo* lipogenesis by altering niacin-induced ERK1/2/AMPK/SIRT1 pathway

Few studies underlined that niacin could inhibit DNL through the activation of extracellular regulated kinase 1/2, AMP-activated protein kinase and sirtuin1 (ERK1/2/AMPK/SIRT1) pathway (20, 32). SIRT1, whose activity is dependent of NAD+ availability, is involved in the transcriptional regulation of *SREBP-1c* and in the epigenetic regulation of *PNPLA3* gene by nutritional factors (33). Therefore, we investigated whether niacin beneficial effects on DNL may be even affected by the loss of PNPLA3 hydrolytic activity.

**FIGURE 6:**
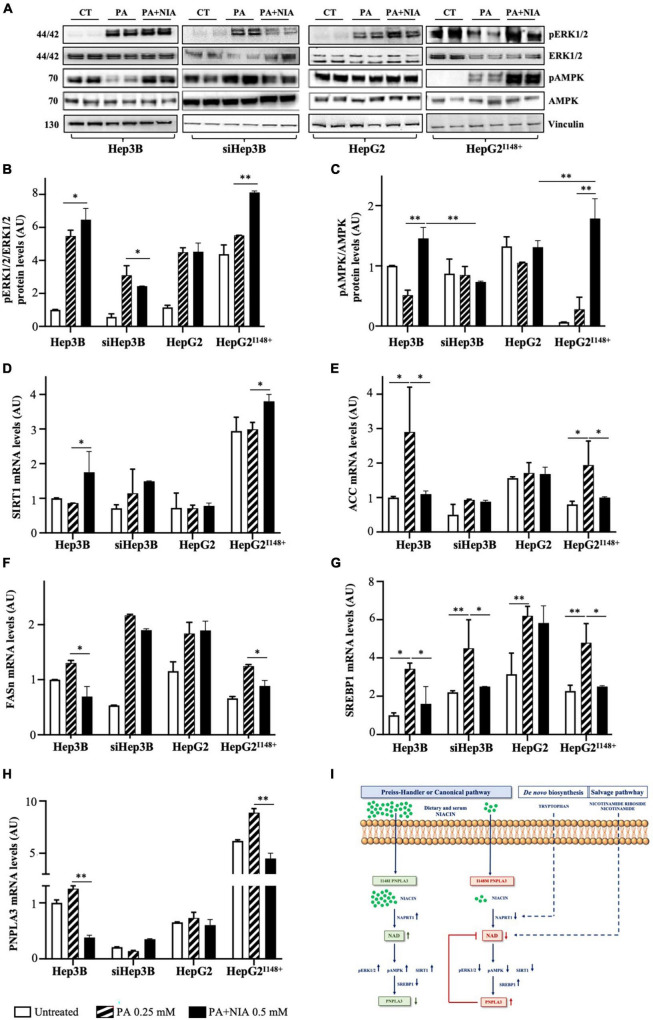
The PNPLA3-niacin crosstalk may occur via ERK1/2/AMPK/SIRT1 pathway. **(A–C)** Phospho-p44/42 MAPK (ERK1/2) (Thr202/Tyr204), p44/42 MAPK (ERK1/2), Phospho-AMPK and AMPK protein levels were evaluated by Western blot in hepatoma cells (Hep3B, siHep3B, HepG2 and HepG2^I148^+^^) after PA challenge and niacin treatment. **(D–H)** *SIRT1*, *ACC, FASn, SREBP1* and *PNPLA3* mRNA levels were assessed in hepatoma cells by qRT-PCR. **(I)** Schematic figure of the putative interplay between the canonical pathway of NAD biosynthesis, which is dependent of niacin availability, and *PNPLA3* genotype. For gene expression, data were normalized to *ACTB* housekeeping gene and expressed as fold increase (Arbitrary Unit-AU) compared to control group. For Western blot, data were normalized to vinculin housekeeping protein and expressed as fold increase (AU) compared to control group. Adjusted *p < 0.05 and ^**^p < 0.01.

In Hep3B and HepG2^I148^+^^ cells, niacin exposure promoted a marked phosphorylation of ERK1/2 (pERK1/2; *p* < 0.05 *vs* Hep3B + PA, *p* < 0.01 *vs* HepG2^I148^+^^ + PA, Figures 6A, B) and AMPK (pAMPK; *p* < 0.01 *vs* Hep3B + PA, *p* < 0.01 *vs* HepG2^I148^+^^ + PA, Figures 6A–C), while they were mildly activated in siHep3B and HepG2 models (Figures 6A–C). Consistently, *SIRT1* mRNA levels were upregulated in Hep3B and HepG2^I148^+^^ cells (*p* < 0.05 *vs* Hep3B + PA, *p* < 0.05 *vs* HepG2^I148^+^^ + PA, Figure 6D), whereas its expression was not significantly changed between siHep3B and HepG2 models at baseline and after treatment with PA or PA + NIA (Figure 6D).

Consequently to the induction of *SIRT1*, Hep3B and HepG2^I148^+^^ cells showed a huge downregulation of genes involved in DNL after niacin administration, as acetyl-CoA carboxylase (*ACC*, *p* < 0.05 *vs* Hep3B + PA, *p* < 0.05 *vs* HepG2^I148^+^^ + PA, Figure 6E), fatty acid synthase (*FASn*, *p* < 0.05 *vs* Hep3B + PA, *p* < 0.05 *vs* HepG2^I148^+^^ + PA, Figure 6F), and *SREBP1* (*p* < 0.05 *vs* Hep3B + PA, *p* < 0.05 *vs* HepG2^I148^+^^ + PA, Figure 6G). In contrast, siHep3B and HepG2 cells showed a mild or no reduction DNL-related genes after niacin exposure (Figures 6E–G), thereby strengthening the theory that niacin efficacy on lipid clearance may be impaired by a non-functional PNPLA3 protein.

In keeping with *SREBP1* downregulation, the mRNA levels of *PNPLA3* were reduced after niacin supplementation in Hep3B and HepG2^I148^+^^ models (*p* < 0.01 *vs* Hep3B + PA, *p* < 0.01 *vs* HepG2^I148^+^^ + PA, Figure 6H) but not in siHep3B and HepG2 ones, corroborating that the possible gene-nutrient interaction between *PNPLA3* and vitamin B3 occurs *via* the ERK1/2/AMPK/SIRT1 signaling and the subsequent *PNPLA3* transcriptional regulation mediated by SREBP1 (Figure 6I).

## Discussion

Environmental risk factors, among which obesity, and genetic variations such as the I148M *PNPLA3* polymorphism strongly contribute to NAFLD pathogenesis. It has been established that PNPLA3 may be modulated by nutrients thus affecting the response to dietary interventions (34, 35). Niacin has been proposed for NAFLD management as it reduces TG synthesis thus improving steatosis in both mice and patients (19, 20). Therefore, we investigated the interplay between the I148M polymorphism and niacin absorption/metabolism in NAFLD patients and *in vitro* models.

We firstly examined food diary in 172 subjects with a non-invasive NAFLD diagnosis (Discovery cohort). We identified a cluster of micronutrients, mostly foods containing fibers and proteins, which appeared less ingested in I148M carriers, thereby offering the possibility to introduce them for a personalized nutritional intervention. Among them, niacin, enriched in fruits, vegetables, meat, and fish, resulted the least consumed. Accordingly, we found that serum niacin was lower in presence of I148M *PNPLA3* variation independently of dietary niacin, suggesting that it may be implied in niacin systemic availability, possibly affecting its absorption and metabolism.

Notably, serum niacin was inversely correlated with body weight and the lowest levels were observed in patients carrying the I148M variant with a BMI ≥ 30 kg/m^2^ both in the Discovery and Validation cohorts, thus supporting a cumulative effect of obesity and the mutation in impairing niacin availability. A relation between niacin intake and BMI changes was previously described by Linder et al. who observed a higher niacin-dependent reduction of hepatic steatosis in NAFLD patients who lost weight during a period of diet counselling and physical activity (25). Consistently, an increased diet-related adiposity was associated with an amplified *PNPLA3* genetic risk of fatty liver. In particular, carbohydrates up-regulate mutant *PNPLA3* on lipid droplets surfaces, thus hindering their hydrolysis (34).

To deepen whether the I148M variant may influence niacin metabolism, we evaluated the hepatic NAD biosynthesis and we found that *NAPRT1*, involved in the canonical NAD pathway, was reduced in subjects with the *PNPLA3* G risk allele independently of steatosis grade, mirroring the low serum niacin levels observed in both Discovery and Validation cohorts. Conversely, the *NADSYN1* and *NMNAT1* mRNA levels, implicated in the alternative and recovery NAD signaling, respectively, increased in I148M carriers as a possible compensatory mechanism to provide hepatic NAD. Alterations of NAD metabolism have been previously associated with NAFLD although no evidence is available regarding the role of genetics in this pathway. According to our findings, Penke et al. have shown that the increased NAD salvage pathway is involved in hepatic steatosis and supplementation with NAD precursors may aid to attenuate disease progression by promoting the NAD^+^-dependent *Sirt1* activation thus highlighting the importance to maintain a sufficient hepatic NAD availability (23).

Our findings in NAFLD patients have suggested that the I148M variation may possibly influence niacin canonical turnover in the liver. To assess the mechanisms through which the PNPLA3 loss-of-function impacts on niacin metabolism, we exploited Hep3B (Wt) and HepG2 (I148M) cells, in which we silenced PNPLA3 or overexpressed the Wt protein, respectively. Hep3B cells exhibited higher levels of NAD which, as expected, increased after niacin exposure. Conversely, siHep3B and HepG2 cells showed lower NAD concentration compared to Hep3B cells and its content did not increase after niacin administration, suggesting an aberrant response to niacin supplementation in presence of the *PNPLA3* loss-of-function. The *PNPLA3* Wt overexpression in HepG2^I148^+^^ model restored both basal and niacin-induced NAD production, suggesting the requirement of a functional PNPLA3 protein to rescue the NAD synthesis. In keeping with the increased NAD levels, niacin exposure promoted the canonical *via* by upregulating *NAPRT1* mRNA in both Hep3B and HepG2^I148^+^^cells, but not in siHep3B and HepG2 ones in which we observed an upregulation of the alternative pathways of NAD biosynthesis, thus resembling the transcriptomic data. In sum, our results have suggested that the PNPLA3 loss-of-function inhibits NAD production from niacin paralleled by the increase of NAD-related alternative pathways.

Niacin can reduce fat accumulation by downregulating TG synthesis *via* DGAT2 in cell cultures, rodents, and humans (24, 36, 37). DGAT2 inhibition was even associated with lower DNL due to less SREPB1-1c nuclear translocation and, consequently, transcriptional regulation of its lipogenic targets, among which *PNPLA3* (21). We found that niacin supplementation improved the intracellular fat content by targeting DGAT2 in all experimental models and it may be due to a combination of reduced TG synthesis and DNL. Such findings were consistent with those reported by Ganji et al. and Blond et al., who demonstrated that niacin treatment directly inhibits DGAT2 activity and reduced TG content in HepG2, HuH7 and primary hepatocytes, and with those of Li et al. who provided the link among DGAT2 inhibition and DNL (21, 24, 36, 37). Another study has pointed out that 39 patients with dyslipidemia improved lipid profile, exhibited lower visceral/subcutaneous fat and ameliorated hepatic fat content after niacin treatment (24, 38). Here, we showed that *PNPLA3* deficiency may affect niacin efficacy on TG synthesis as the siHep3B and HepG2 cells displayed less reduction of hepatocellular TG compared to Hep3B and HepG2^I148^+^^ models.

Furthermore, it has been demonstrated that niacin reverses oxidative stress and inflammation, in cells and animal models, possibly due to its effect in clearing lipids (38). We observed greater ER-oxidative stress in siHep3B and HepG2 cells than Hep3B and HepG2^I148^+^^ models after PA challenge, likely exacerbated by the presence of a non-functional PNPLA3 protein. Another mechanism by which niacin could reduce fat accumulation foresees the activation of the ERK1/2/AMPK/SIRT1 signaling (32, 39). Several studies revealed that phospho-AMPK and treatments with NAD^+^ precursors promote *SIRT1* activation, which, in turn, decrease DNL (22, 23, 32, 39). Ye et al. have demonstrated that niacin treatment in obese HFD-fed mice hampered the transcriptional activity of *SREBP1* through ERK1/2/AMPK activation with the consequent reduction of hepatic and plasma TG content (40). We found that niacin promoted the ERK1/2 and AMPK phosphorylation paralleled by SIRT1 mRNA upregulation more in Hep3B and HepG2^I148^+^^ rather than siHep3B and HepG2 cells, suggesting that the PNPLA3 loss-of-function may reduce the inhibition of DNL induced by niacin. Consistently, niacin promoted a significant reduction of *SREBP1, ACC* and *FASn* expression in Hep3B and HepG2^I148^+^^ cells, but not in siHep3B and HepG2 models. Recently, it has been proven that the AMPK/SIRT1 may hamper the SREBP1 binding to PNPLA3 promoter (33) and as above mentioned SREBP1 is involved in the transcriptional regulation of PNPLA3 after carbohydrates loading (34). We found that *PNPLA3* mRNA levels were decreased in Hep3B and HepG2^I148^+^^ after niacin supplementation, and this effect may be ascribable to the low *SREBP1* expression. Conversely, niacin did not alter *PNPLA3* expression in HepG2 cells possibly suggesting that the crosstalk between PNPLA3 and niacin may occur through SREBP1.

To date, niacin was proposed at pharmacologic doses in the range of 1,500–2,000 mg (Niaspan^®^) for the treatment of dyslipidemia and prevention of cardiovascular complications (37). A clinical trial carried out in 39 hyper-triglyceridemic patients with steatosis has showed a reduction of liver fat by 47% and liver enzymes when treated with Niaspan for 6 months (37).

Lifestyle interventions have shown a great efficacy to improve hepatic steatosis and they currently represent a valid approach for NAFLD management. Preclinical studies have demonstrated that dietary intake of NAD precursors may ameliorate fatty liver by boosting the hepatic NAD metabolism. Accordingly, our study revealed a novel nutrigenetic regulation of the *PNPLA3* gene by niacin and underlined how the genetic screening which is useful in terms of costs and non-invasiveness gains value for a personalized approach in NAFLD patients. By looking at a translational perspective, either the nutritional supplementation with niacin or the increased consumption of niacin-fortified foods may represent an alternative option to overcome the low niacin levels observed in genetically predisposed NAFLD patients, even more with the co-occurrence of obesity.

## Conclusions

The I148M *PNPLA3* variant, the strongest genetic predictor of NAFLD onset and progression, may undergo nutritional regulation and its deleterious effect could be worsened by environmental factors as obesity. Niacin, belonging to Vitamin B class, has been suggested for NAFLD management as it reduces hepatic fat content and inflammation. In this study, we highlighted a potential interaction between the presence of mutant PNPLA3 and niacin metabolism. Dietary evaluation through food diary pointed out that NAFLD subjects, among which around 60% carried the I148M variant and > 40% was affected by obesity, exhibited an unbalanced diet with low intake of vitamins, fibers, and proteins. In patients, our results have supported that the PNPLA3 CG/GG genotype was associated with lower niacin availability in the serum and, at hepatic levels with altered expression of enzymes involved in NAD biosynthesis promoting more the alternative pathways than the canonical *via*. Even in hepatocytes, the presence of PNPLA3 loss-of-function limited the NAD production through the canonical pathway after niacin supplementation as well as it dampened niacin efficacy on fat accumulation and oxidative stress, thus sustaining the possible gene-nutrient crosstalk. In sum, vitamin B3 supplements or niacin-fortified foods should be recommended for NAFLD patients with a predisposing genetic background, amplified by adiposity.

## Data availability statement

The ethical approval of the study does not allow to publicly share individual patients’ genetic data. All data, code and materials used in the analysis are available upon reasonable request for collaborative studies regulated by materials/data transfer agreement (MTA/DTA) to the corresponding author.

## Ethics statement

The studies involving human participants were reviewed and approved by Fondazione IRCCS Cà Granda CE: 164_2019. The patients/participants provided their written informed consent to participate in this study.

## Author contributions

EP and ML: study design, data analysis and interpretation, and manuscript drafting. MMe: data analysis and interpretation and manuscript drafting. GT: data analysis. AC, RL, SB, and MMa: patients recruitment and characterization. AF: discussion and manuscript revision. PD: study design, manuscript drafting, data analysis and interpretation, study funding, supervision, and has primary responsibility for final content. All authors read and approved the final manuscript.

## Footnotes

1
https://www.meteda.it/en/product/metadieta
