# Supplementary material for: The I148M PNPLA3 variant mitigates niacin beneficial effects: How the genetic screening in non-alcoholic fatty liver disease patients gains value
Source: Front Nutr. 2023 Mar 2;10:1101341. doi: 10.3389/fnut.2023.1101341 (PMC10018489; doi:10.3389/fnut.2023.1101341)
Supplement: Supplementary file 1 [file Data_Sheet_1.pdf]

The I148M PNPLA3 variant mitigates Niacin beneficial effects: how the genetic screening in Non-alcoholic Fatty Liver Disease (NAFLD) patients gains value

Running title: PNPLA3-niacin nutrigenomic interplay in NAFLD

Erika Paolini[1,2*], Miriam Longo[1,3*], Marica Meroni[1], Giada Tria[1], Annalisa Cespiati[1,4], Rosa Lombardi[1,4], Sara Badiali[5], Marco Maggioni[6], Anna Ludovica Fracanzani[1,4], Paola Dongiovanni[1]

[1]*General Medicine and Metabolic Diseases, Fondazione IRCCS Ca' Granda Ospedale Maggiore Policlinico, Pad. Granelli, via F Sforza 35, 20122 Milan, Italy;* [2]*Department of Pharmacological and Biomolecular Sciences, Università degli Studi di Milano, Milano 20133, Italy;* [3]*Department of Clinical Sciences and Community Health, Università degli Studi di Milano, Milano 20122, Italy;;* [4]*Department of Pathophysiology and Transplantation, Università degli Studi di Milano, 20122 Milano, Italy;* [5]*Surgery, Fondazione IRCCS Ca' Granda Ospedale Maggiore Policlinico, 20122 Milan, Italy;* [6]*Pathology, Fondazione IRCCS Ca' Granda Ospedale Maggiore Policlinico, 20122 Milan, Italy.*

**These authors equally contribute to the paper.*

Correspondence to: Dr. Paola Dongiovanni, General Medicine and Metabolic Diseases, Fondazione IRCCS Ca' Granda Ospedale Maggiore Policlinico, Pad. Granelli, via F Sforza 35, 20122 Milan, Italy. paola.dongiovanni@policlinico.mi.it; Tel.: +39-02-5503-3467; Fax: +39-02-5503-4229. ORCID: 0000-0003-4343-7213

Supplemental materials and methods

Chemicals

Bovine serum albumin (BSA) and Oil Red O were purchased from Sigma-Aldrich (St Louis, MO). Anti-P-p44/42 MAPK, anti-p44/42 MAPK (ERK1/2) antibodies, anti-IgG mouse and anti-IgG rabbit were acquired from Cell Signaling Technologies (Boston, United States). Anti-vinculin was purchased from Abcam (Cambridge, UK). Anti-mGFP and pLenti-C-mGFP lentiviral vector were obtained from OriGene (Rockville, MD). Dulbecco's modified Eagle's medium (DMEM), fetal bovine serum (FBS), phosphate-buffered saline (PBS), L-Glutamine, penicillin/streptomycin, Trypsin/EDTA, Hank's balanced salt solution (HBSS), lipofectamine 3000 transfection reagent, SuperScript VILO cDNA Synthesis Kit, fast SYBR green master mix, and TaqMan Fast Universal PCR master mix 2x were obtained Life Technologies-ThermoFisher Scientific (Waltham, United States).Clarity western ECL substrate was obtained from Bio-Rad Laboratories (Hercules, United States). NAD/NADH Colorimetric Assay Kit (ab65348), Triglycerides Colorimetric/Fluorimetric Assay Kit (ab65336), Hydrogen Peroxide Assay Kit (ab102500), Lipid Peroxidation (MDA) Assay Kit (colorimetric/fluorometric) (ab118970), were bought from Abcam (Cambridge, UK). VectaMount AQ Mounting Medium was obtained from Maravai Life Sciences Inc. (United Kingdom).

Transcriptomic analysis

RNAseq was performed in a subset of 183 severely obese patients (31 without and 152 with NAFLD) of whom percutaneous liver biopsy was performed during bariatric surgery at Fondazione IRCCS Cà Granda, Ospedale Policlinico, Milan, Italy and transcriptomic data was available. The study was conformed to the Declaration of Helsinki and approved by the Institutional Review Boards and their Ethics Committees. All participants gave written informed consent. Clinical characteristics of the Transcriptomic cohort are presented in **Table S5**.

Total RNA was isolated using RNeasy mini-kit (Qiagen, Hulsterweg, Germany), according to the manufacturer's instructions. RNA quality was assessed through Agilent 2100 Bioanalyzer and samples with RNA integrity numbers (RIN) greater than or equal to 7 were used for library preparation. RNA sequencing was performed in paired-end mode with a read length of 150nt using the Illumina HiSeq 4000 (Novogene, Hong Kong, China). Illumina raw reads were mapped against the Human Genome [1] using a custom pipeline based on the standard primary analysis procedure. The pipeline performed the primary analysis step including FASTQ quality check (FastQC software, Babraham Bioinformatics, Cambridge, UK), low-quality reads trimming with Trimmomatic [2] and mapping on GRCh37 reference genome using STAR mapper [3]. RNASeq quality control was performed, and samples with less than 10 million reads uniquely mapped or with less than 60%

uniquely mapped/mapped reads were excluded from the analysis. Gene reads count was performed using RSEM software [4]. To quantify gene expression RSEM per-gene counts were normalized using DESeq2 package [5].

Histologic Evaluation

The severity of disease in the Validation cohort and Transcriptomic cohort was assessed through the liver biopsy. Steatosis was divided into the following 4 categories based on the percentage of affected hepatocytes: 0, 0%–4%; 1, 5%–32%; 2, 33%–65%; and 3, 66%–100%. Disease activity was assessed according to the NAFLD activity score (NAS), encompassing the evaluation of hepatocellular ballooning and necroinflammation; fibrosis also was staged according to the recommendations of the NAFLD Clinical Research Network [6].

Genotyping

The Discovery, the Validation and the Transcriptomic cohorts were genotyped for the *PNPLA3* rs738409 C>G (p.I148M) variant[7-9]. Genotyping was performed in duplicate using TaqMan 5'-nuclease assays (QuantStudio 3, Thermo Fisher, Waltham, MA). Results of rs738409 genetic frequency was compared to those obtained in not-Finnish European healthy individuals included in the 1000 Genome project[10]. Genotyping success rate was >99%.

Oil Red O (ORO) staining

Cells were plated on 6-wells plate ($5x10^5$ cells/well) in duplicate and left overnight in DMEM medium containing 10% FBS, 1% L-glutamine and 1% Penicillin/Streptomycin. After 24 hours, grow media was removed and cells were kept 24h in quiescent medium, containing 0.5% BSA, 1% L-glutamine and 1% Penicillin/Streptomycin. The day after, we performed Oil Red O (ORO) staining, which is a soluble red powder with high affinity for neutral triglycerides (TG) and lipids stored in the lipid droplets (LDs). Quiescent medium was removed, and the 6-well plates were gently rinsed with 2 mL of sterile PBS 1X. Next, cells were fixed with 4% formalin for 15 minutes at room temperature. After fixation, each sample was washed with sterile water and 60% isopropanol was added for 5 minutes. ORO working solution was prepared by mixing 3 parts of ORO stock solution (300 mg of Red Oil powder in 100 mL di isopropanol 100%) and 2 parts of sterile water, following filtration. Each sample was incubated with ORO working solution for 45 minutes. Hematoxylin was used to counterstain the nucleus. LDs content appeared in pink-red color.

Gene expression analysis

RNA was extracted from cell cultures using Trizol reagent (Life Technologies-ThermoFisher Scientific, Carlsbad, U.S.A). 1µg of total RNA was retro-transcribed with VILO random hexamers

synthesis system (Life Technologies-ThermoFisher Scientific, Carlsbad, U.S.A). Quantitative real time PCR (qRT-PCR) was performed by an ABI 7500 fast thermocycler (Life Technologies), using the TaqMan Universal PCR Master Mix (Life Technologies, Carlsbad, CA) and TaqMan probe for human *PNPLA3* (ThermoFisher #Hs00403495_m1). The SYBR Green chemistry (Fast SYBR Green Master Mix; Life Technologies) was used for all other genes. All reactions were delivered in triplicate. Data were normalized to the β-actin (*ACTB*) gene expression and results were expressed as arbitrary units (AU) or fold increase as indicated in bar graphs. Primers are listed in **Table S6.**

Western Blot Analysis

Total protein lysates were extracted from cell cultures, using RIPA buffer containing 1 mmol/L Na-orthovanadate, 200 mmol/L phenylmethyl sulfonyl fluoride and 0.02 μg/μL aprotinin. Samples were pooled prior electrophoretic separation and all reactions were performed in duplicate. Then, equal amounts of proteins (30 μg) were separated by SDS-PAGE, transferred electrophoretically to nitrocellulose membrane (BioRad, Hercules, CA) and incubated with specific antibodies overnight. At least, three independent lots of freshly extracted proteins were used for experiments. Antibodies and concentration used are listed in **Table S7**.

Supplementary Tables

Table S1. List of micro- and macro-nutrients (kcal) obtained from the food diary compiled by the Discovery cohort (n=172) and calculated through MètaDieta software

	Discovery cohort (n=172)		†P value
	CC	CG/GG	
**Alcohol (g)**	50.65±10.10	34.16±6.87	0.05
**Proteins (g)**	729.6±55.72	555.9±42.11	0.07
**Lipids (g)**	433.5±33.20	372.5±30.77	0.19
• Saturated fats	141.6±13.01	153.6±12.12	0.94
• Monounsaturated fats	166.1±13.83	171.8±12.23	0.15
• Polyunsaturated fats	73.40±6.65	65.73±4.36	0.05
• Cholesterols (mg)	2148±189.5	2063±169.7	0.05
**Carbohydrates (g)**	1144±84.77	1179±68.49	0.87
• Starch	571.8±45.93	641.5±42.44	0.70
• Insoluble sugars	474±46.69	426.5±27.82	0.52
• Fructose	65.63±9.71	46.82±6.11	0.37
**Fibers (mg)**	186.31±118.87	151.24±77.43	**0.04**
• Sodium	8381±715.9	9335±886.1	0.99
• Potassium	18471±1752	14435±975.3	**0.03**
• Iron	530.8±55.87	543.7±55.84	0.55
• Calcium	5875±581.2	5346±425.7	0.35
• Phosphorus	12079±1071	10570±625.9	0.13
• Vit. A ret.	6723±749.9	5656±452.5	0.13
• α-carotene	111.6±36.74	84.38±14.03	0.57
• β-carotene	31981±4050	25822±2592	0.09
• Thiamin (Vit. B1)	271.9±34.36	316.7±47.04	0.43
• Riboflavin (Vit. B2)	29.10±3.80	22.96±2.33	0.06
• **Niacin (Vit.B3)**	142.9±13.66	116±7.97	**0.04**
• Vit. C	1567±187.7	1275±98.88	0.15
• Vit. E	25.63±5.57	23.55±2.46	0.09
• Vit. D (mcg)	65.63±9.71	103.2±25.58	0.31
• Polyphenols*	2529±348.2	1728±269.1	0.06
• Polyphenols**	17949±2637	12683±1310	0.05
**Total Fibers (g)**	187.4±20.36	154.2±11.38	0.06
**Total kcal (T-kcal)**	11869±857	11866±704.2	0.36
• Alcohol	351.3±72.53	238.6±48.99	**0.04**
• Proteins	2954±223.1	2654±161	**0.04**
• Lipids	3916±298.9	3978±272.5	0.24
• Carbohydrates	4479±354.2	4718±274	0.79
• Fibers	375±40.73	308.4±22.75	**0.04**
**kcal/day**	1696±122.4	1695±100.6	0.36
• Alcohol	50.21±10.35	34.09±6.99	**0.04**
• Proteins	422±31.86	379.2±23	**0.04**
• Lipids	559.4±42.7	568.3±38.94	0.24
• Carbohydrates	639.9±50.6	674±39.13	0.79
• Fibers	53.63±5.82	44.11±3.24	**0.04**
**% kcal/T-Kcal**			
• Alcohol	2.85±0.59	1.73±0.27	**0.04**
• Proteins	24.33±0.71	22.96±0.62	0.11
• Lipids	31.98±0.90	32.67±0.76	0.68
• Carbohydrates	7.92±1.25	40.02±1.34	0.79
• Fibers	3.15±0.20	2.92±0.18	**0.04**

Values have been reported as average ± standard deviation (SD). *Polyphenol compounds (mg) assessed by Liquid Chromatography–Tandem Mass Spectrometry Technique (LC–MS/MS). **Total polyphenols assessed by Folin & Ciocalteu′s' reagent. The cluster of micronutrients less consumed by carriers of the rs738409 C>G PNPLA3 variant is highlighted in light grey. Niacin remains statistically significant in subjects carrying the rs738409 C>G PNPLA3 variant and with steatosis≥2 (†$p<0.05$ CC vs CG/GG).

Table S2. List of dietary products containing niacin (mg) obtained from the food diary

	Food ration (g)	Niacin (mg)
**Fruits**		4.37
• Orange	150	0.30
• Banana	150	1.05
• Pear	100	0.10
• Grape	150	0.60
• Melon	200	1.20
• Hazelnut	40	1.12
**Vegetables**		18.95
• Lentils	60	0.36
• Zucchinis	100	11
• Grean beans	200	1.60
• Artichokes	100	150
• Cucumber	40	0.24
• Carrots	50	0.35
• Broccoli	150	1.50
• Salad Tomatoes	200	1.40
• Tomato sauce	250	2
**Cereals and derivatives**		2.39
• Bread	90	0.91
• Pasta	120	0.72
• Rice	60	0.24
• Mais	50	0.43
• Breadsticks	10	0.09
**Meat and derivatives**		20.61
• Beef	120	5.64
• Pork	120	4.80
• Bologna	30	1.08
• Sausange	70	3.57
• Baked ham	60	1.84
• Raw hame	60	3.30
• Eggs	120	0.36
**Fish and derivatives**		33.16
• Tuna in oil	120	12.48
• Sardines	200	19.40
• Mussels	80	1.28
**Milk and derivatives**		2.21
• Full-fat Milk	150	0.15
• Mozzarella	100	0.40
• Provola	100	0.40
• Grana	80	0.08
• Fontina	80	0.16
• Gorgonzola	80	0.72
• Soft cheese	100	0.30
**Snacks and bakery**		2.33
• Pizza	100	2.10
• Chocolate	60	0.23
**Beverage**		7.59
• Caffè	80	4.17
• Birra	330	2.97
• Juices	150	0.45

Table S3. Demographic, anthropometric, and clinical features of NAFLD patients (n=172, Discovery cohort)

	**Discovery cohort (n=172)**
Sex, M	128 (74.4)
Age, years	52.44±12.15
BMI, kg/m^2	29.27±4.43
Obesity, yes	74 (43.02)
IFG/T2D, yes	25 (14.53)
HOMA-IR	3.84±4.30
Insulin, IU/mL	17.28±16.27
Total cholesterol, mmol/L	5.28±0.19
LDL cholesterol, mmol/L	2.07±0.14
HDL cholesterol, mmol/L	1.67±0.11
Triglycerides, mmol/L	4.85±0.49
ALT, IU/L	9.27 {9.02-9.62}
AST, IU/L	3.25 {3.04-3.61}
Steatosis ≥2	114 (66.27)
Dietary niacin, kcal	4.69±0.61
Serum niacin, µg/µL	4.91±0.51
PNPLA3 I148M, yes	104 (60.46)

Values have been reported as average ± standard deviation (SD), number (%) or median and interquartile range {IQR}, as appropriate. BMI: body mass index; IFG: impaired fasting; T2D: type 2 diabetes mellitus**.** Variables with skewed distribution were logarithmically transformed.

Table S4. Demographic, anthropometric, and clinical features of biopsied NAFLD patients (n=358, Validation cohort)

	**Validation cohort (n=358)**
Sex, M	214 (59)
Age, years	49.41±12.98
BMI, kg/m^2	32.38±7.31
Obesity, yes	194 (54.18)
IFG/T2D, yes	113 (31.5)
HOMA-IR	6.22±8.33
Insulin, IU/mL	24.12±23.24
Total cholesterol, mmol/L	4.98±1.17
LDL cholesterol, mmol/L	3.13±1.04
HDL cholesterol, mmol/L	1.25±0.34
Triglycerides, mmol/L	1.70±1.02
ALT, IU/L	3.82 {3.29-4.30}
AST, IU/L	3.46{3.13-3.82}
Steatosis ≥2	232 (64.80)
Serum niacin, µg/µL	5.23±0.82
PNPLA3 I148M, yes	255 (71.2)

Values have been reported as average ± standard deviation (SD), number (%) or median and interquartile range {IQR}, as appropriate. BMI: body mass index; IFG: impaired fasting; T2D: type 2 diabetes mellitus**.** Variables with skewed distribution were logarithmically transformed.

Table S5. Demographic, anthropometric, and clinical features of 183 severely obese patients of whom RNA-seq data were available.

	**Normal liver (n=31)**	**Mild NAFLD (n=97)**	**Severe NAFLD (n=55)**	***P value***
Sex. F	29 (94)	88 (91)	34 (62)	**<0.0001**
Age, years	41.2 ± 8.1	42.8 ± 11.1	44.7 ± 9.2	0.28
BMI, kg/m^2	37.4 ± 8.6	41.2 ± 6.2	43.2 ± 7.7	**0.002**
IFG/T2D, yes	1 (3.5)	10 (11)	9 (15.4)	0.25
Total cholesterol, mmol/L	5.8 ± 1.3	5.5 ± 1.0	5.1 ± 1.2	0.10
Triglycerides, mmol/L	1.1 ± 0.4	1.5 ± 0.7	1.7 ± 1.0	**0.01**
LDL cholesterol, mmol/L	3.4 ± 0.8	3.5 ± 0.8	3.1 ± 1.0	0.09
HDL cholesterol, mmol/L	1.6 ± 0.3	1.4 ± 0.3	1.3 ± 0.3	**0.002**
ALT, IU/L	16 [13-22]	20 [16-26]	31 [23-43]	**<0.0001**
AST, IU/L	16 [13-20]	17 [15-22]	24 [18-31]	**<0.0001**

Values are reported as mean ± SD. number (%) or median {IQR}. as appropriate. BMI: body mass index; IFG: impaired fasting; T2D: type 2 diabetes mellitus**.**

Table S6. Sequence of primers used in quantitative real-time PCR experiments.

	Forward 5'→3'	Reverse 5'→3'
ACC	CAGAGATGTTTGCGCAGTCA	GGCTACCATGCCAATCTCAT
ATF4	AAACCTCATGGGTTCTCCAG	GGCATGGTTTCCAGGTCACT
ATF6	AATTCTCAGCTGATGGCTGT	TGGAGGATCCTGGTGTCCAT
FASn	GCTGGGTGGAGTCTCTGAAG	TGCAACACCTTCTGCAGTTC
GRP78	CTTGCCGTTCAAGGTGGTTG	CTGCCGTAGGCTCGTTGAT
NADSYN1	CTGCCTCATCTACTCCATGTGC	GGTGTAGCTGATCTGGTTCACG
NAPRT1	AGCCACGAATGAAGCTGACCGA	CACTGGCTCTTCTGCTAACTGC
NMNAT1	GTGGAAAGAGACTCTGAAGGTGC	CTTGTGTTTCAGTCCACTTCCTC
PNPLA3	TTCAGAGGCGTGCGATATG	TCTCTCCCAGCACCTTGAGA
SIRT1	TAGACACGCTGGAACAGGTTGC	CTCCTCGTACAGCTTCACAGTC
β-actin	GCTACAGCTTCACCACCACA	AAGGAAGGCTGGAAAAGAGC

Table S7. List of antibodies and relative dilutions used in Western blot.

Antibody	Catalog Number
mGFP (1:1000 WB)	OriGene # TA180076S
p-p44/42 MAPK (ERK1/2) (1:1000 WB)	Cell signaling #9101S
p44/42 MAPK (ERK1/2) (1:1000 WB)	Cell signaling #9102S
pAMPK	Cell signaling #2531S
AMPK	Cell signaling #2532S
Vinculin (1:1000 WB)	Abcam #ab73412

Supplementary Figures

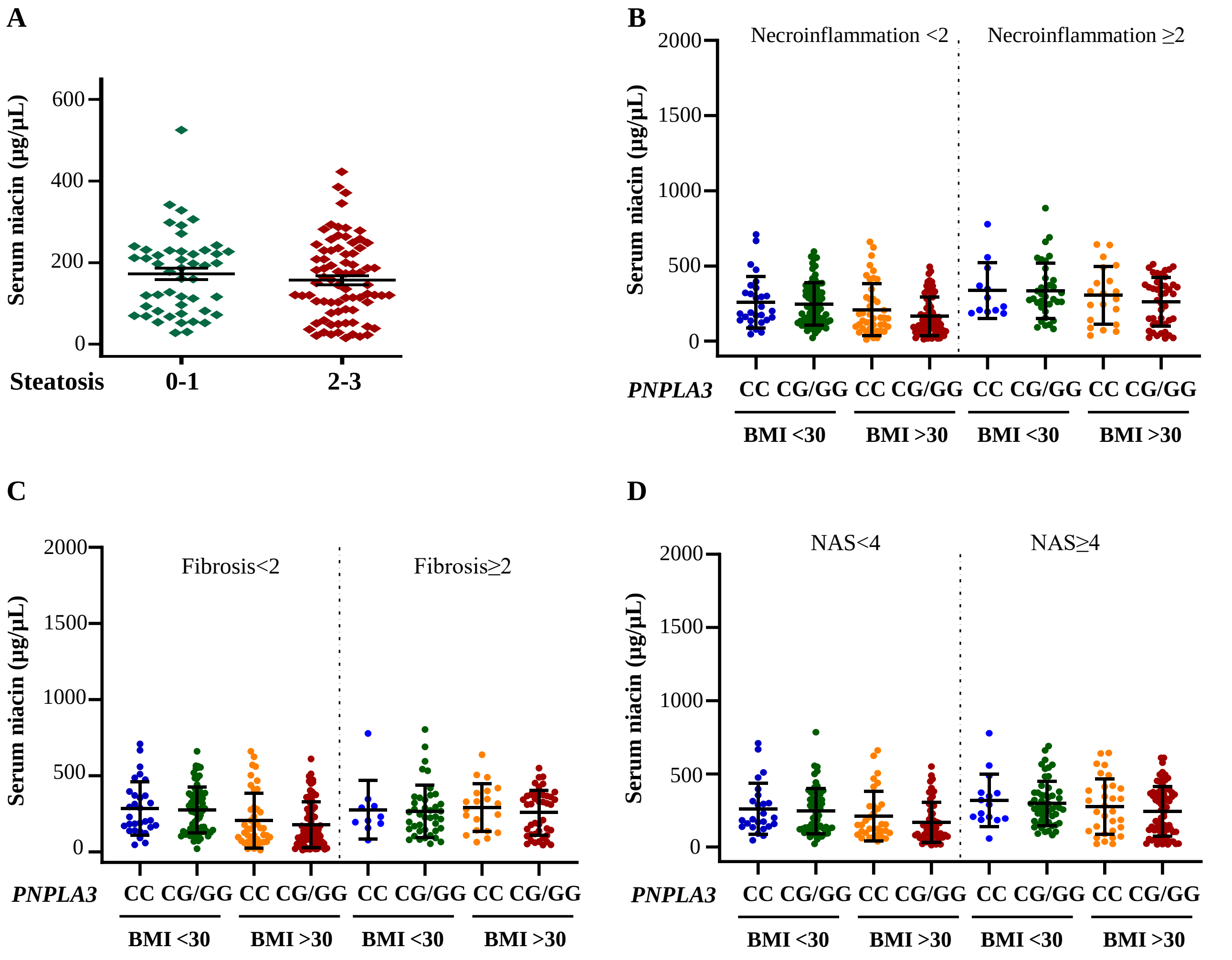

Figure S1: NAFLD severity did not influence systemic niacin availability. **A**) Circulating niacin levels were slightly reduced in NAFLD patients with steatosis ≥2 (Discovery cohort). **B-D**) Bivariate analysis shows a non-significant correlation between serum niacin levels and severity necroinflammation, fibrosis and NAFLD activity score (NAS) in biopsied NAFLD subjects of the Validation cohort.

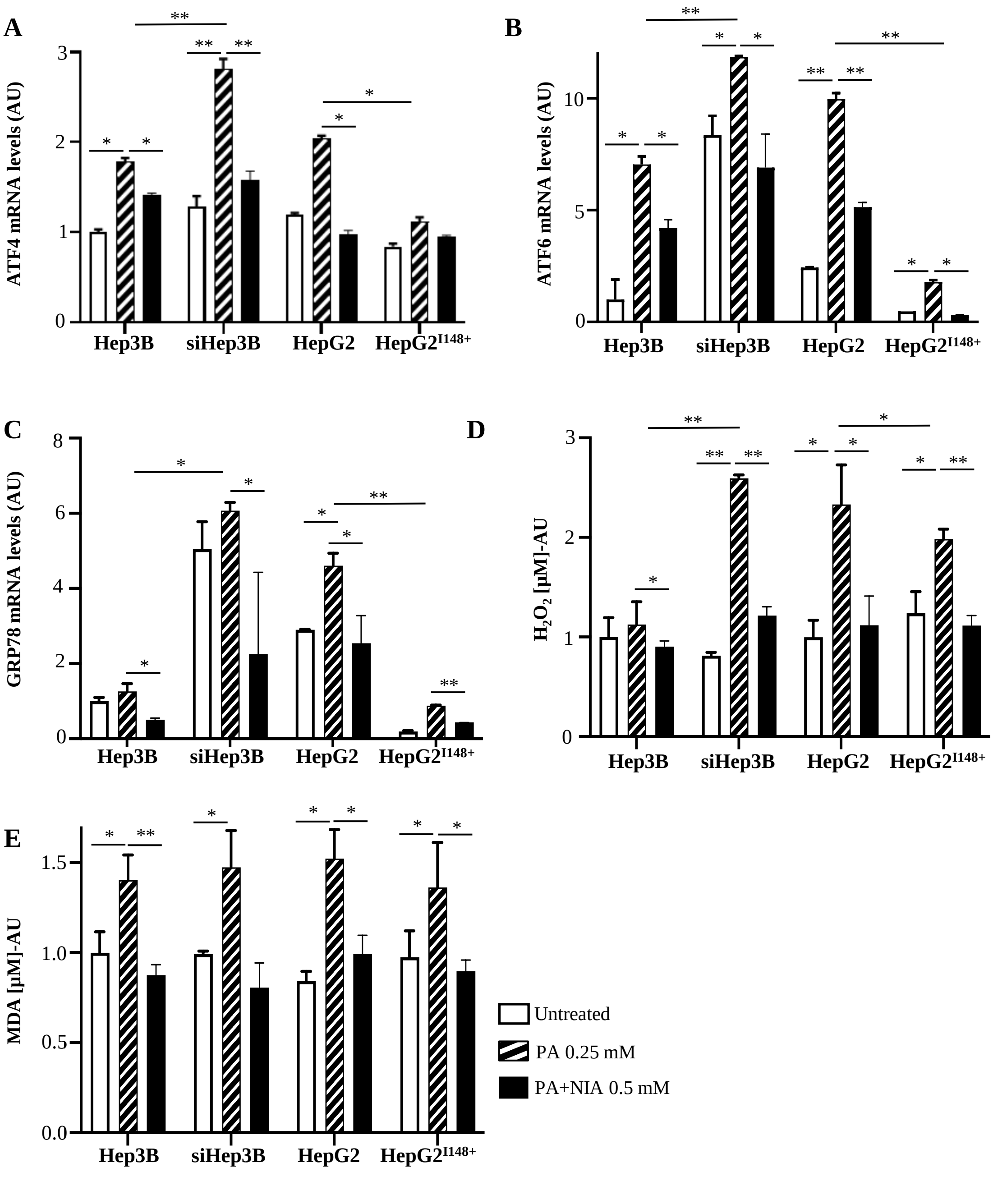

Figure S2: Niacin reduces ER/oxidative stress independently of the PNPLA3 loss-of-function. **A-C**) ATF4/6, and GRP78 mRNA levels were measured in hepatoma cells (Hep3B, siHep3B, HepG2 and HepG2I148+) with or without niacin treatment after PA challenge by qRT-PCR. **D**) H2O2 was quantified in Hep3B and siHep3B cell lysates before or after niacin exposure by a Hydrogen Peroxide Assay Kit Colorimetric Assay Kit (Abcam, Cambridge, UK). **E**) The malondialdehyde (MDA) was colorimetrically measured in Hep3B, siHep3B, HepG2 and HepG2I148+ cell lysates by Lipid peroxidation assay kit (Abcam, Cambridge, UK). For gene expression, data were normalized to ACTB housekeeping gene and expressed as fold increase (Arbitrary Unit-AU) compared to control group. Adjusted *p<0.05 and **p<0.01

References

1. Cunningham, F., et al., *Ensembl 2015.* Nucleic Acids Res, 2015. **43**(Database issue): p. D662-9.
2. Bolger, A.M., M. Lohse, and B. Usadel, *Trimmomatic: a flexible trimmer for Illumina sequence data.* Bioinformatics, 2014. **30**(15): p. 2114-20.
3. Dobin, A., et al., *STAR: ultrafast universal RNA-seq aligner.* Bioinformatics, 2013. **29**(1): p. 15-21.
4. Li, B. and C.N. Dewey, *RSEM: accurate transcript quantification from RNA-Seq data with or without a reference genome.* BMC Bioinformatics, 2011. **12**: p. 323.
5. Love, M.I., W. Huber, and S. Anders, *Moderated estimation of fold change and dispersion for RNA-seq data with DESeq2.* Genome Biol, 2014. **15**(12): p. 550.
6. Kleiner, D.E., et al., *Design and validation of a histological scoring system for nonalcoholic fatty liver disease.* Hepatology, 2005. **41**(6): p. 1313-21.
7. Meroni, M., et al., *Mboat7 down-regulation by hyper-insulinemia induces fat accumulation in hepatocytes.* EBioMedicine, 2020. **52**: p. 102658.
8. Longo, M., et al., *TM6SF2/PNPLA3/MBOAT7 Loss-of-Function Genetic Variants Impact on NAFLD Development and Progression Both in Patients and in In Vitro Models.* Cellular and Molecular Gastroenterology and Hepatology.
9. Meroni, M., et al., *MAFLD definition underestimates the risk to develop HCC in genetically predisposed patients.* 2022. **291**(3): p. 374-376.
10. McVean, G.A., et al., *An integrated map of genetic variation from 1,092 human genomes.* Nature, 2012. **491**(7422): p. 56-65.
